# Insights From Veterinary Disciplinary Actions in California 2017–2019

**DOI:** 10.3389/fvets.2021.786265

**Published:** 2021-12-23

**Authors:** Jake Labriola, Rebecca Garabed, Carly Sinclair, Antoinette E. Marsh

**Affiliations:** ^1^Department of Veterinary Preventive Medicine, College of Veterinary Medicine, The Ohio State University, Columbus, OH, United States; ^2^Moritz College of Law, The Ohio State University, Columbus, OH, United States

**Keywords:** veterinary disciplinary actions, standard of care, California Veterinary Medical Board, sanctions, medical records

## Abstract

There is increasing concern within the veterinary medical community (veterinarians and veterinary students) that disgruntled clients are unfairly leveraging various legal tools against veterinarians. Clinical veterinarians and veterinary students should be aware of the most common types of problems arising within the clinic and how they can lead to formal consumer complaints. The study describes and categorizes with greater detail the types of violations or “causes for discipline” that occur, as well as specific sanctions imposed on veterinarians formally disciplined for standard of care-related violations between 2017 and 2019, for California. In addition, the study calculated the frequency of disciplinary actions and their basic summary statistics regarding the temporal aspect of how lawsuits typically unfold. Using public documents from California, the study describes the analysis and trends for the purpose of providing contextual evidence to inform and guide potential veterinary educational interventions. Although specific to California, this study can serve as a template methodology for comparisons to other states.

## Introduction

Increasingly, Americans view pets as important members of their social in groups rather than expendable property (1). Nonetheless, animals are treated as property in all 50 states in the United States (2). The increased social status of pets is reflected by the rapidly growing pet industry, which reached over $62 billion in 2016 (3). In 2018, the American Veterinary Medical Association (AVMA) estimated there are over 76 million pet dogs and over 58 million pet cats in the United States (4). By some estimates, about 75% of Americans' pets regularly sleep on the bed with their owner and millions of pets have their own social media accounts (5). Not surprisingly, people have been increasingly willing to spend significant amounts of money to purchase the best veterinary care possible (6). Further proof of owner's willingness to spend money on veterinary care is the insurance industry marketing policies specifically to cover veterinary care and the rise in owners who have purchased pet insurance policies (7). One potential consequence resulting from the transformation of pet social status is a perceived increase in legal actions taken by pet owners dissatisfied with their pet's medical care (6).

The AVMA made significant progress toward achieving a nationally unifying framework to hold veterinarians accountable to a common standard of care when the Model Veterinary Medicine Practice Act was first passed in 1964 (8). Nonetheless, standards of veterinary care are still not nationally consistent. Most legal licensing-related actions against practicing veterinarians occur at the state level *via* the individual state's Veterinary Practice Acts (VPAs) and the state's Veterinary Medical Boards (VMBs). State VMBs oversee the practice of veterinary medicine to ensure both consumers and veterinarians are protected from, for example, substandard care or unsupported consumer complaints, respectively. VMBs and VPAs also ensure that the licensed veterinarian continues to uphold the Veterinarian's Oath. If a formal complaint is received, the state VMB uses its discretion whether to investigate and determine if disciplinary legal action is justified in accordance with state-specific laws. Disciplinary sanctions resulting from legal action also vary by state and can have serious adverse consequences for veterinarians, such as license revocation, probation, fines, and required continuing education (6).

There is increasing concern within the veterinary medical community (veterinarians and veterinary students) that disgruntled clients are unfairly leveraging various legal and social media tools against veterinarians (9). Clinical veterinarians and veterinary students should be aware of the most common types of problems arising within the clinic and how they can lead to formal consumer complaints (9). Through increased awareness and education, it may be possible to reduce anxiety of veterinarians who do their best to diagnose and treat diseases, particularly when the patient outcome is unfavorable despite appropriate and documented efforts. Furthermore, veterinarians should be aware of what sanctions (the money and time) are commonly imposed by a state VMB when a violation is found in the clinic or consequentially from the veterinarian's actions or inactions. While informal consumer complaints through social media can have devastating social, emotional, and financial consequences for veterinarians, only formal complaints to VMBs result in formal review and sanction. Thus, while both types of complaints have consequences, only formal complaints that the VMBs determine are valid violations are systematically documented such that we can assess violations of standard of care and proportional consequences of those, filed with the VMB. Therefore, the focus of this study is only on the disciplinary action against the veterinarian after a VMB pursued a complaint deemed a violation. The study did not evaluate “social media” or internet ranking or complaints against veterinarians.

Babcock et al. (9) attempted to identify the major type of infraction per disciplinary action against licensed veterinarians in 10 states from 2005 to 2011 and found that professional negligence or malpractice was by far the most common type of infraction. The definitions and inclusion criteria for each disciplinary action category were not consistent in that study, presumably due to state-to-state variation and temporal changes in veterinary laws. In contrast, a generalized definition of professional negligence is a breach of the standard of care and is defined as “the veterinarian failed to use such reasonable skill, diligence, and attention as may ordinarily be expected of careful, skillful and trustworthy persons in the profession” (10). In cases for boarded specialist, these individuals are held to a higher standard relative to their advanced training in their specialty area. The standard for administrative discipline is language that is set out in the disciplinary codes, so there could be some variation between professional negligence and disciplinary code as the former generally uses expert witness testimony to establish through a court proceedings whereas administrative disciplinary actions are initially handled differently. Disciplinary action cases cannot simply be lumped into a single infraction category, especially when there are multiple causes for discipline per disciplinary action case. To illustrate this issue, consider the differences between two states Ohio and California. While the Ohio VMB separately lists “standard of care” violations from “record keeping” violations, the California VMB encompasses “record keeping” violations within the parent category of “standard of care” violations.

Considering the differences in definitions to be a major limitation of the Babcock et al. (9) study, our study sought to narrow the scope of our investigation to the state level. Our study describes and categorizes with greater detail the types of violations or “causes for discipline” that occur, as well as specific sanctions imposed on veterinarians formally disciplined for standard of care-related violations between 2017 and 2019, for California. In addition, the study calculated the frequency of disciplinary actions and their basic summary statistics regarding the temporal aspect of how lawsuits typically unfold. Using public documents from California, the study describes the analysis and trends for the purpose of providing contextual evidence to inform and guide potential veterinary educational interventions. Although specific to California, this study can serve as a template methodology for comparisons to other states.

## Materials and Methods

### Sample Population

To obtain the data from formally disciplined veterinarians, a California Public Records Act (CPRA) request was performed on November 7, 2019, and approved by the California State VMB on November 15, 2019. The list we received was composed of veterinary license numbers for all veterinarians whose disciplinary action cases contained both a “Final Decision” and an “Accusation rooted in a sub-standard of care argument” between November 7, 2017 and November 7, 2019. Note that this population only includes disciplined veterinarians and not consumer complaints or investigations that failed to meet the VMB's criteria to pursue disciplinary actions. Consumers can initiate a complaint using the Department of Consumer Affairs, Veterinary Medical Board Consumer Complaint Form (Supplementary Materials).

### Data Collection and Digital Organization

The list of veterinary license numbers was then used to identify and collect publicly available disciplinary case documents, *via* the License Lookup Portal tool built into the BreEZe database located within the California Department of Consumer Affairs (CA DCA) website. The disciplinary case documents collected consisted of two main types, *Accusations* or *Decision and Order*. These documents concern the bulk of the research focus and therefore constitute the primary data source used in this study.

All documents were downloaded, renamed according to case number and document type, and then uploaded to a secure, cloud-based working folder. Renamed files were structured systematically, so they could be precisely indexed within their associated folders. The case-retrieval document containing the list of veterinary license numbers was also stored within the digital database.

### Data Extraction and Validation

A comprehensive content review of five randomly selected cases was conducted to gain familiarity of legal document structure and to establish a preliminary data extraction procedure. Variable data were extracted directly from these documents and transferred to a novel spreadsheet, with individual veterinary disciplinary cases (the unit of analysis) along the vertical axis. Data from three variables (Trigger Event Date, Violations, and Zip Code) were extracted from the *Accusation* documents, while data from the remaining variables was extracted from the *Decision and Order* documents (Served Date, Order Date, License Revocation Status, Stayed Revocation Status, License Suspension Status, License Suspension Duration, Probation Status, Probation Duration, Restitution Status, Restitution Amount, Fine Status, Fine Amount, Cost Recovery Amount, Continuing Education (CE), Ethics Training (ET), Community Service (CS), Clinical or Written Exam Status, Psychological Evaluation Status, and Drug or Alcohol Rehabilitation Status). Five variables (Notification Period, Litigatory Phase, Total Resolution Time, Total Time Cost, and Total Monetary Cost) were derived secondarily. In other words, data for each variable was populated systematically as case content was reviewed. All case-related data was cleaned and cross-verified by another researcher to mitigate any potential data extraction inconsistencies.

### Time-Interval Data

Three distinct time intervals were calculated from dates extracted from documents. The first time period is described as the *Notification Period*, which indicates the number of days between the initial *Trigger* event and the day when the veterinarian was legally served notice of disciplinary action. Our definition of a *Trigger* event requires that the *Accusation* document included an incident date, incident location, and an objective description of an incident that ultimately led to a consumer complaint or unannounced premises inspection. The second time interval calculated is described as the *Litigatory Phase*. This was calculated by subtracting the *Served* date from the *Order* date. This interval captures the number of days between the veterinarian's notice of indictment and the exact date on which the VMB reached a disciplinary conclusion called the *Decision and Order*. The third time interval is described as the *Total Resolution Time*. *Total Resolution Time* was calculated by subtracting the initial *Trigger* event date from the *Order* date.

### Sanctions Data

Sanctions imposed by the California VMB can be characterized into three categories, License-related, Time-Commitment, or Monetary. These three categories were then further described in terms of severity or frequency of occurrence. License-related sanctions include the following variables: Revocation Status, Suspension Status, Suspension Duration, Probation Status, and Probation Duration. Time-commitment sanctions involved the following variables: Continuing Education Status, Ethics Training Status, Community Service Status, Continuing Education Amount, Ethics Training Amount, Community Service Amount, and Continuing Education Topics. Monetary sanctions variables included the following: Restitution Status, Fine Status, Restitution Amount, Fine Amount, and Cost Recovery Amount.

### Geographic Data

Zip code data was extracted from *Accusation* documents to identify and depict the geographic locations where disciplinary actions in our sample area arose. ESRI's ArcMap software (https://www.esri.com/en-us/home) was used to create a map document showing the spatial distribution of zip codes involved with the study.

### Incidence Data

A second California Public Records Act request was performed on August 10, 2020, to obtain the annual number of consumer complaints against veterinarians, in addition to the annual number of active state veterinary licenses during the years 2017, 2018, and 2019. The California Department of Consumer Affairs responded by providing these data timely. Incidence rates were calculated for each individual year, as well as the 3-year average incidence rate to help understand the risk of veterinarians receiving a consumer complaint filed to the CA state VMB against their active license. This is distinguished from the remainder of the study where we are looking at only those complaints that result in disciplinary action.

### Violation Data

The *Causes for Discipline* or *Statutory Violations* for each case were extracted from the *Accusation* and recorded in the profile for each case. The study focused on identifying and describing potential trends related to veterinary standard of care violations and their associated disciplinary sanctions. A tabulated data set was created for each disciplinary action case. RStudio Desktop (https://www.rstudio.com/products/rstudio/) was used to import, visualize, and describe the distribution of all quantitative variables in terms of basic descriptive statistics, such as central tendency and variance. For each disciplinary action case, the total number of Causes for Discipline, or Violations, was tabulated. From 59 veterinary disciplinary action cases reviewed, the study calculated the median, maximum, and minimum number of violations per disciplinary action case. A similar approach was taken with the imposed sanctions (license suspension, probation term, ethics training, community service, continuing education, and fines) along with the restitution and cost recovery. When the data was normally distributed a mean rather than median was reported. The resulting data was plotted using a box plot of the 25–75% interquartile range with whiskers to show minimum and maximum, and also inclusion of any outliers to the interquartile range on the displayed plots.

## Results

Throughout the 3-year retrospective study period from 2017 to 2019, the average incidence of formal complaints against veterinarians was ~7 complaints per 100 active veterinary licenses (Table 1). Specifically, complaints received by the Board against licensed veterinarians were the following 905 in 2017; 844 in 2018 and 936 in 2019. The data does not show an obvious increase or decrease in incidence from year to year within this period. Essentially, the incidence rate appears to be constant. Throughout the study period, there were about 13,000 active veterinary California licenses. Of these, only 59 veterinarians were disciplined by the State VMB.

**Table 1:** New complaints to the Veterinary Medical Board from 2017 to 2019, number of active Veterinary licenses within California, and the calculated incident rate per 100 active licenses.

**Risk of receiving a consumer complaint for licensed veterinarians in CA**				
**Year/time period**	**2017**	**2018**	**2019**	**2017–2019**
New Complaints Filed To VMB	905	844	936	2,685
Active Veterinary Licenses	12,749	13,057	13,001	38,807
Incidence Rate per 100 Active Licenses	7.1	6.5	7.2	6.9

While all 59 of the veterinarians in the sample had their licenses revoked, this revocation was stayed for all but 16 veterinarians. In other words, 27% of formally disciplined veterinarians had their license outright revoked by the State of California. For the remaining 43 veterinarians (73%) who had their revocation stayed, this meant their revocation could be lifted if they fulfill various probationary contingencies.

**Figure 1:**
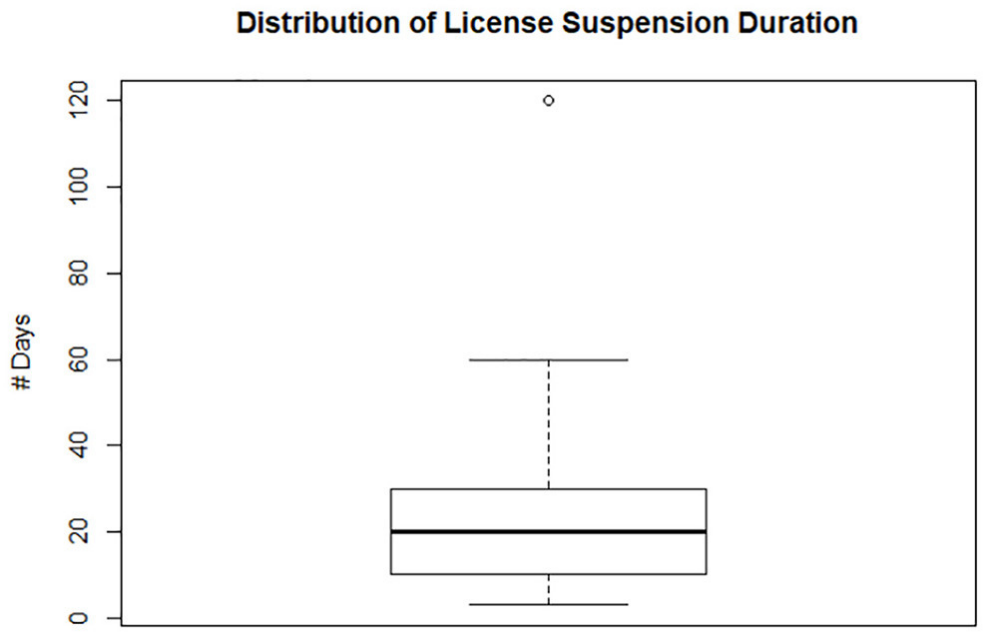
Box plot for days of license suspension imposed on 20 veterinarians, ranged from 60 to 3 days with the median of 20 days. One outlier received 120 days.

**Figure 2:**
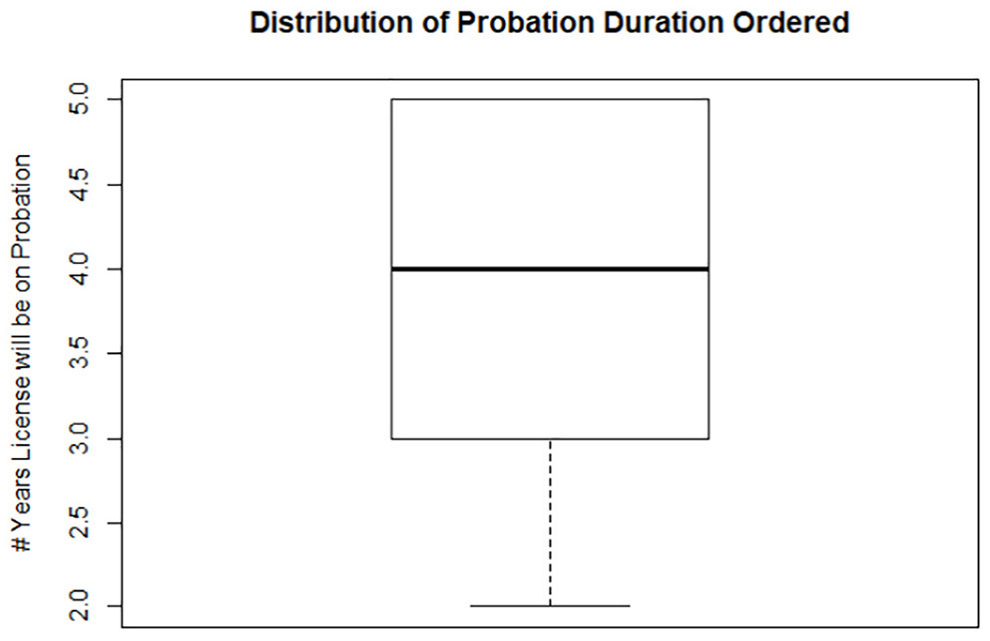
Box plot of probation term for 43 veterinarians whose licenses were placed on probation status which ranged from 5 to 2 years, with the median 4 years.

Of the 43 veterinarians who had their licenses stayed, contingent upon the conditions of probation, 23 veterinarians had their license suspended (made inactive) for a duration of time. For the 23 veterinarians who had their license suspended before they could start their probationary period, the median number of days their licenses were suspended was 20 days. The maximum number of suspension days was 120 days, while the minimum number of days was 3 days (Figure 1). Furthermore, those 43 veterinarians whose licenses were placed on probation, the median duration of probation was 4 years, the minimum number was 2 years, and the maximum number required was 5 years (Figure 2).

**Figure 3:**
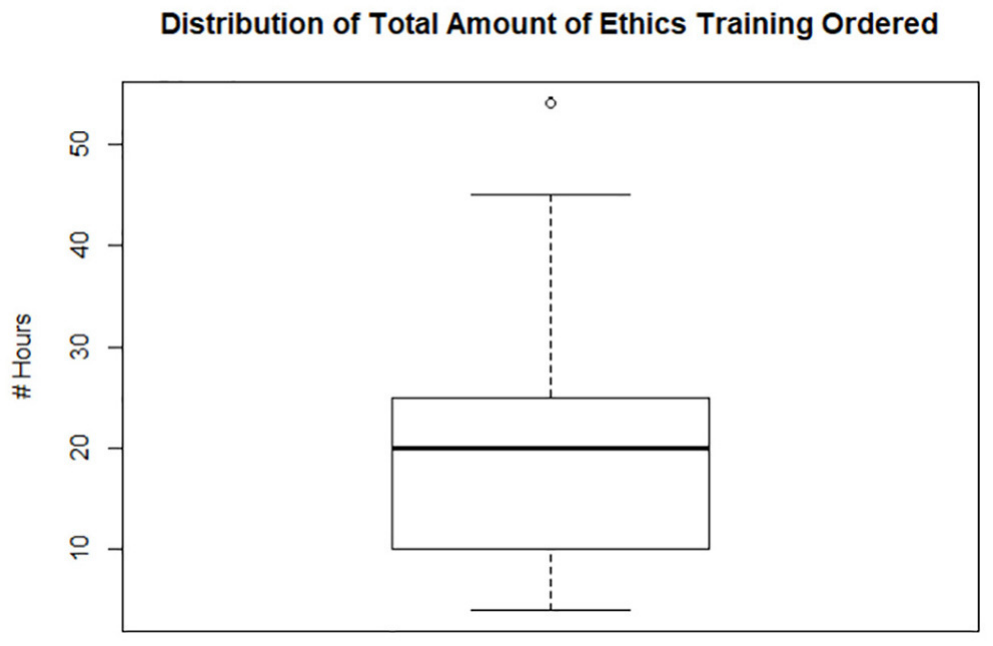
Box plot of ethics training imposed on 25 of 43 veterinarians whose licenses were placed on probation, which ranged from 45 to 4 h, with 20 h as the median number of hours required. One outlier received 54 h.

**Figure 4:**
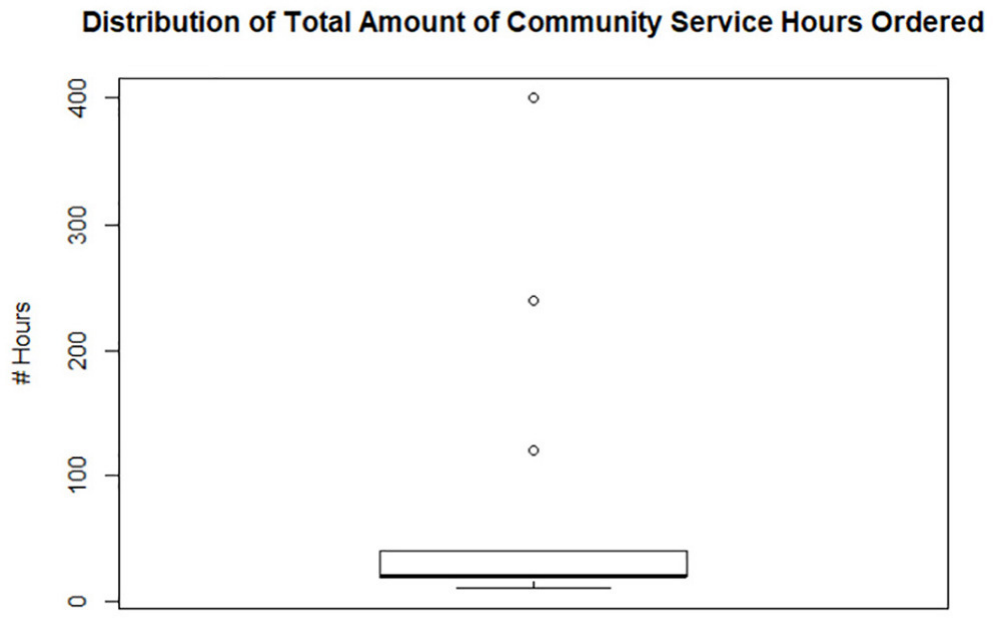
Box plot of community service imposed on 14 of 43 veterinarians whose licenses were placed on probation which ranged from 40 to 10 h with 20 h as the median number of hours required. Three outliers received >100 h with one receiving 400 h.

**Figure 5:**
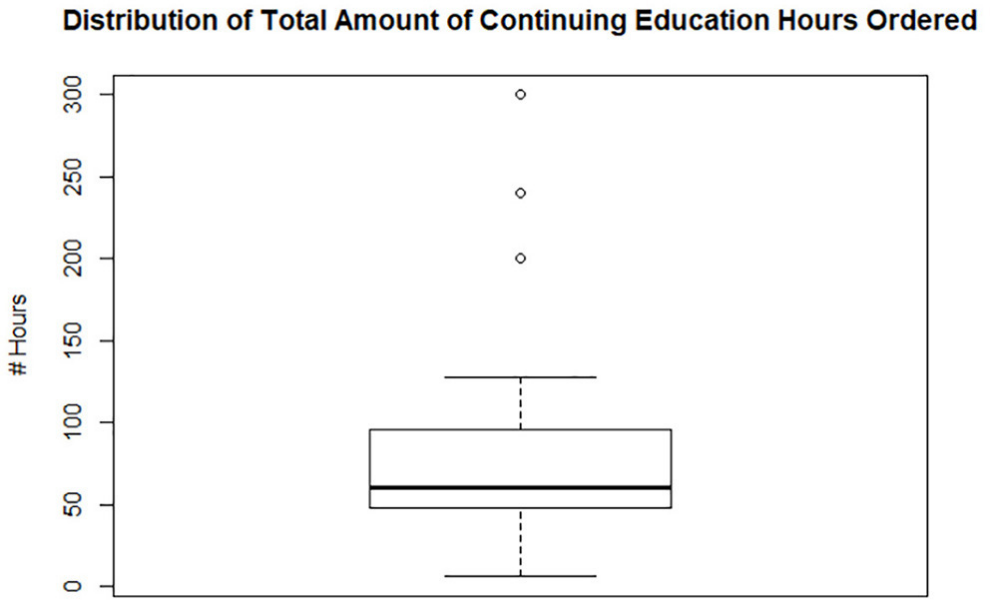
Box plot of continuing education imposed on 41 of 43 veterinarians whose licenses were placed on probation which ranged from 128 to 6 h with 60 h as the median number of hours required. Three outliers received ≥200 h with one receiving 300 h.

Of the 43 licenses placed on probation, 14 veterinarians received community service mandates, 25 received ethics training mandates, and 41 received continuing education mandates. The median number of hours imposed for those who received community service, ethics training, and continuing education, was 20, 20, and 60 h, respectively. There were 3 outliers within the continuing education group, which skewed the data's calculated mean. There were also 3 outliers within the community service group, which skewed the mean higher (Figures 3–5).

**Figure 6:**
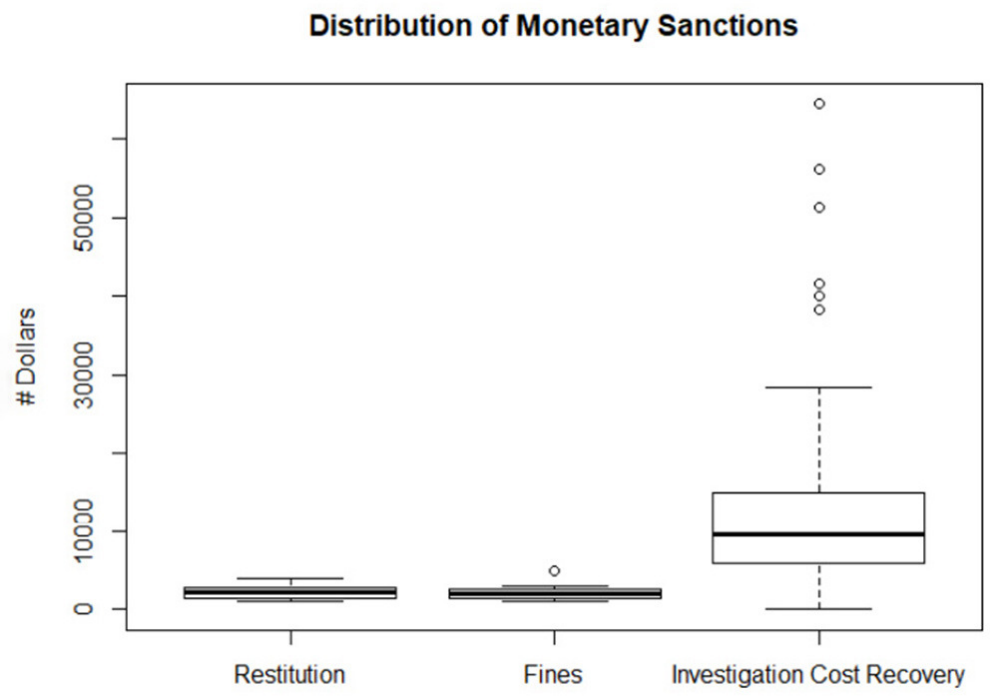
Box plot of restitution, fines and investigation costs are shown. Restitution (*n* = 11) imposed which ranged from $3960 to $1000 with the mean of $2,213; Fines (*n* = 33) imposed ranged from $5,000 to $1,000 with the mean of $2,424; 3 outlier incurred a fine of $5000 (dots are superimposed); Cost recovery (*n* = 58) ranged from $64,456 to $888 with the median fine of $9,600. Six outliers exceeded $30,000 in cost recovery. A single case with no cost recovery is not included.

All veterinarians were required to pay the cost recovery for the investigation, excluding one veterinarian who paid $0. The median number of dollars veterinarians were required to pay for cost recovery was $9,600. The maximum amount was $64,456, while the minimum (excluding the veterinarian who did not have to pay the cost recovery) was $888. Of the 11 veterinarians required to pay restitution, the mean was $2,213, the maximum was $3,960, and the minimum was $1,000. Of the 33 veterinarians required to pay a fine, the mean was $2,424, the maximum was $5,000, and the minimum was $1,000 (Figure 6). These values were normally distributed without any outliers; therefore, a mean value is used to describe and represent the data. Nonetheless, these financial figures do not include the veterinarian's legal or professional fees paid by the veterinarian or their insurance company for the defense or legal advice relating to the VMB's disciplinary action.

Evaluation of the time-interval data found that the mean number of years for the notification period, the litigatory phase, and the total resolution was ~3, 1, and 4 years, respectively.

From the sample of 59 veterinary disciplinary action cases, the study calculated the median, maximum, and minimum number of violations per disciplinary action case (5, 41, and 1, respectively). The top three most common types of violations were professional negligence (76%), records-related (66%), and incompetence (46%). Although many of the violations and decision orders were detailed and specific to instances of violations, some general examples provide guidance on behaviors or actions that should be avoided.

For example, under a records-related violation, the veterinarian did not keep any medical records whatsoever for an unknown number of patients. When an unsatisfied owner requested medical records, the veterinarian failed to provide any documents to the client, and then the client proceeded to file a complaint with the VMB. When the veterinarian was subpoenaed by the VMB, the veterinarian fraudulently forged medical records to submit for review.

As an example of incompetence violation, one veterinarian spayed a service dog in estrus and left the dog alone in a cage to recover overnight, and the dog was found deceased the next morning. A necropsy revealed the animal died from uncontrolled intra-abdominal hemorrhage, secondary to inadequate ligation of an ovarian pedicle.

Finally, in one case involving professional negligence, a veterinarian anesthetized a cat prior to performing dental extractions and then the veterinarian left the surgery area. After waiting 20 min to start the procedure, a technician found the veterinarian unresponsive from a drug overdose in the apartment located above the clinic.

**Figure 7:**
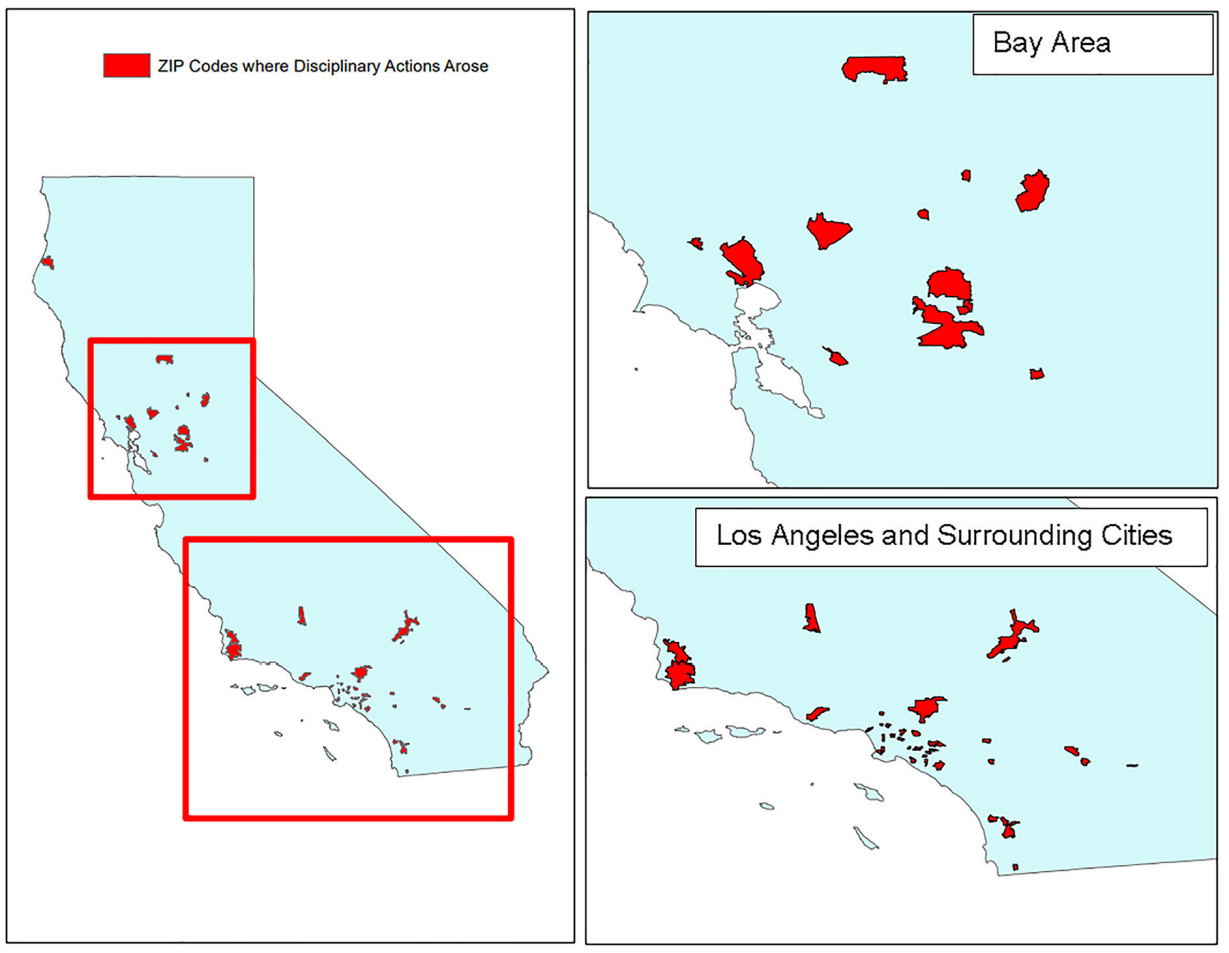
California geographic zip code location mapped by incident where disciplinary action arose with the distribution and density clustering plotted in red. Distribution tends to be in urbanized areas with few to no disciplinary actions in the agricultural, less populated areas, such as the San Joaquin central valley or north central California.

Some notable patterns were identified from our statistical analyses. All VMB disciplinary actions involved feline, canine or a combination of these species. Two disciplinary actions also included pet porcine as patients. Surprisingly, no disciplinary cases involved client complaints with horses or livestock. The zip code analysis suggests that urbanized areas with greater population density experienced a greater number of disciplinary actions (Figure 7). However, this may also be reflective of a greater number of veterinarians serving the population.

## Discussion

Overall, complaints to the CA VMB are very rare with only 7 in 100 active licenses having complaints filed. Our sample (*N* = 59) consisted of all Disciplinary Actions rooted in a standard of care argument that conclusively reached a Final Decision and Order between the years 2017 and 2019 in the State of California, which represents <5 cases per 1,000 active licenses. The data represented veterinarians who had at least one complaint filed to the VMB which ultimately resulted in a VMB disciplinary action with at least one or more sanctions. One important limitation to understand regarding the data and analysis in this study is that our statistical results do not represent all complaints filed against the veterinarians during this period nor all the details from the subsequent investigations. Rather, the data is from complaints and investigations within the VMB public documents which were used to support the disciplinary measures imposed by the VMB. Therefore, consumer complaints or investigations that failed to meet the VMB's criteria to pursue disciplinary actions were not included in the in-depth study. Informal complaints and complaints without sufficient justification for disciplinary action are more common, but difficult to study systematically.

Our data is important to demonstrate the California VMB's consistency with the imposed sanctions (license suspension, probation term, ethics training, community service, continuing education, and fines) and restitution and cost recovery related to the disciplinary action. In the box plotted data, the few outliers beyond the maximum range represent veterinarians with more egregious and intentional acts such as fraudulently altering the medical record, obtaining blood from euthanized or dying patients without client consent, not cross matching or testing the blood for blood borne diseases, or falsifying health certificates. Additionally, prior citations from the VMB were noted in the findings and were considered aggravating elements.

However, this study revealed that the topic of veterinary disciplinary action related to standard of care violations is extremely nuanced and would be extremely challenging to assess/categorize at the national level until appropriate methods for state-level analyses are established. The terms, documentation components, and code sections used by state VMBs can vary between states. One question our study sought to answer was whether this approach can be scaled-up to compare state differences, and we found creating a uniform objective data entry spreadsheet was challenging to create when comparing records from California to Ohio for those reasons listed as well as available access to the public records during COVID-19 pandemic imposed closures. Moreover, to directly compare California and Ohio, some subjective interpretation of matching code sections was required; therefore, the analysis was not pursued. Until a national legal framework is created, this seems unlikely. Because the AVMA's Model Veterinary Medicine Practice Act merely provides unifying recommendations to state VMBs, the legal framework for veterinary law will continue to be extremely variable from state to state, preventing state-to-state comparisons from being possible or appropriate.

Approximately 66% of all cases cited professional negligence related to record keeping. It is not surprising that about 44% of all cases requiring probationary conditions also included mandates to fulfill *record keeping* continuing education requirements. One hypothesis that could explain why *record keeping* violations and related sanctions were common findings, is that medical records are subpoenaed legal documents which can serve as hard, indisputable, time-stamped evidence of professional negligence in an otherwise “factually” weak disciplinary argument. In other words, there might have been more serious violations involved in a disciplinary case, but there was no or insufficient evidence to support prosecution of those violations.

Other notable patterns described concerns the time-interval data and the time duration required for these cases to typically unfold and come to a final decision regarding the sanction. The implications of the longer-than-expected *Notification Period* highlights that veterinarians might not know for several years that their actions/behaviors might become subject to disciplinary action(s). The *Litigatory Phase* duration suggests that once a veterinarian is legally served, a disciplinary order (when justified) is mandated more quickly (i.e., within about 1 year). However, it should be noted that the true amount of time involved in a disciplinary action case is much longer, since probation can last for many years beyond sentencing or if a veterinarian seeks to appeal the VMB finding then it will also result in a longer case duration. The study did not track the fulfillment status or sanction completion of veterinarians on disciplinary probation, and this additional time was not included in this study's time-interval analysis. The *Total Resolution Time* suggests that the overall litigative process is lengthy, often lasting more than 4 years.

Perhaps the most meaningful conclusion from this study is that more stringent and consistent record keeping behaviors could confer significantly increased legal protection from disciplinary actions. While there is a potential for veterinarians to spend too much effort on record keeping such that they diminish their ability to provide care, our analysis suggests that legal action still relates more toward lack of records than over-recording. More research regarding record keeping as a potential target for educational intervention is needed. As the field of veterinary medicine continues to evolve we expect record keeping and documentation to become more important, especially in the areas of antimicrobial use, pandemic-related waivers for telemedicine,^1^ and continuing education, and all of these changes involve documentation and record keeping. Therefore, the importance of consistent record keeping cannot be understated if veterinarians and veterinary students want to avoid legal action and be prepared for the changes ahead.

## Data Availability Statement

The raw data supporting the conclusions of this article will be made available by the authors, without undue reservation.

## Author Contributions

The project was conceived by AM and RG. AM prepared the funding proposal. JL and RG designed the data entry and subsequent analysis. JL and CS collected and collated the results. The manuscript was prepared by JL and reviewed by all authors with significant editorial assistance from AM.

## Funding

Support for this study was provided by the Stanton Foundation, a private foundation with canine health and welfare as one of its primary missions.

## Conflict of Interest

The authors declare that the research was conducted in the absence of any commercial or financial relationships that could be construed as a potential conflict of interest.

## Publisher's Note

All claims expressed in this article are solely those of the authors and do not necessarily represent those of their affiliated organizations, or those of the publisher, the editors and the reviewers. Any product that may be evaluated in this article, or claim that may be made by its manufacturer, is not guaranteed or endorsed by the publisher.

## Footnotes

^1^California Veterinary Medical Board voted to extend COVID-19 pandemic associated-telemedicine waivers (condition specific Veterinarian-Client-Patient Relationship and prescription refills) until the pandemic is over. This suggests the rules will change in the future.

## Supplementary Material

Supplementary Data 1 Spreadsheet with cases with data categories.

Supplementary Data 2 Department of Consumer Affairs, Veterinary Medical Board, Consumer Complaint Form.
