# Supplementary material for: Insights From Veterinary Disciplinary Actions in California 2017–2019
Source: Front Vet Sci. 2021 Dec 23;8:786265. doi: 10.3389/fvets.2021.786265 (PMC8734430; doi:10.3389/fvets.2021.786265)
Supplement: Supplementary Data 1 — Spreadsheet with cases with data categories. [file Data_Sheet_1.PDF]

Order_Reviewed	Zip_Incident_Location	Animals_Involved	Initial_Event
1	92201	1 Dog	9/8/2014
2	90025	1 Dog	10/24/2013
3	92707	3 Dogs, others (unknown)	4/1/2010
4	90038	1 Cat and 10+ Dogs	4/21/2015
5	92037	3 Dogs, 1 Cat	4/20/2015
6	90241	1 Dog	11/8/2014
7	93436	1 Dog	9/13/2012
8	93455	1 Dog	8/31/2012
10	92647	1 Dog	2/4/2012
11	92618	1 Dog	1/6/2014
12	92064	1 Dog, 1 Cat	11/8/2013
13	90501	1 Dog	9/24/2014
14	93012	1 Dog, 1 Cat	3/2/2015
15	95682	10 Dogs, 5 Cats	7/6/2013
16	95476	2 Dogs, 1 Cat	3/20/2015
17	92801	1 Cat	12/3/2014
18	90623	1 Dog	7/24/2015
19	92867	3 Dogs, others	6/14/2010
20	90505	1 Dog	10/6/2015
21	92586	None	2/9/2016
22	95242	1 Dog	1/26/2016
23	91504	3 Dogs	12/22/2009
24	91915	1 Dog, 2 Cats	5/6/2010
25	95953	1 Dog	3/14/2016
26	92276	2 Dogs	7/7/2016
32	92025	same as case 26, but the father's fol	7/7/2016
27	90640	4 Dogs	6/5/2009
28	91702	1 Dog	3/25/2016
29	92078	10+ Dogs, 10+ Cats	10/24/2016
30	94583	1 Dog, 4 Cats	5/12/2015
31	92886	1 Dog	7/17/2017
33	95503	2 Dogs	5/30/2009
34	95206	7 Dogs, 1 Cat	1/29/2012
35	92706	2 Dogs	8/12/2003
36	92887	2 Dogs	10/15/2014
37	95207	10+ Dogs, 10+ Cats (records violatio	6/17/2015
38	95621	1 Cat, 2 Dogs	12/18/2009
39	90026	1 Cat	7/29/2012
40	92379	1 Cat, 1 Dog	9/22/2011
41	95355	1 Cat, 3 Dogs	8/6/2009
42	90255	2 Dogs	10/1/2011
43	93313	1 Dog	11/12/2012

44	92553	1 Cat, 1 Dog	9/25/2015
45	95688	The only animals mentioned were for	7/1/2013
46	94928	None	12/3/2014
47	95831	2 Dogs (plus 10+ cats/dogs mentione	8/23/2013
48	90404, 90272	1 Cat, 1 Dog, Humans Involved	4/5/2006
49	95210	2 Dogs	8/3/2010
50		2 Dogs	10/8/2013
51		1 Cat, 1 Dog	1/9/2015
52		1 Dog	2/27/2015
53		1 Dog, 1 Pig	8/8/2014
54		1 Dog	10/22/2013
55		1 Dog	3/2/2014
56		2 Dogs	5/14/2007
57	91745	1 Dog	1/26/2016
58	92311	9 Dogs, 1 Pig	2/15/2014
59	90047	1 Dog, 1 Cat related to consumer cor	2/25/2016
60	85083	1 Dog	3/2/2016

Initial_Event_Type	First_Accusation_Served	Order_Date
Pet visit	5/1/2018	10/24/2019
Pet visit	9/30/2016	11/6/2017
Inspection	4/21/2014	10/14/2014
Pet visit	3/18/2019	5/20/2019
Controlled Substance Order	9/26/2016	10/31/2017
Pet visit	4/26/2016	5/2/2017
Pet visit	3/1/2016	11/29/2017
Pet visit	11/9/2015	2/24/2017
Pet visit	10/13/2015	4/13/2017
Pet visit	3/23/2016	4/11/2017
Pet visit	10/16/2015	11/1/2016
Pet visit	8/4/2016	8/8/2017
Pet visit	10/24/2017	4/24/2019
Pet visit	4/19/2018	2/8/2019
Pet visit	3/9/2017	6/5/2018
Pet visit	3/8/2018	5/21/2019
Pet visit	10/30/2017	10/3/2018
Pet visit	10/11/2016	8/1/2017
Pet visit	5/19/2017	8/16/2018
Inspection	1/23/2017	6/22/2017
Pet visit	8/14/2017	7/20/2018
Pet visit	10/22/2013	5/4/2015
Pet visit	12/15/2015	7/23/2018
Pet visit	9/14/2017	3/8/2018
Pet visit	5/30/2018	7/26/2019
Pet visit	6/4/2018	3/18/2019
Pet visit	2/2/2012	2/4/2014
Pet visit	9/1/2017	10/3/2018
Inspection	8/24/2018	8/26/2019
Pet visit	9/19/2017	4/16/2019
Pet visit	2/14/2019	9/18/2019
Pet visit	3/11/2013	6/16/2014
Pet visit	4/5/2016	3/9/2018
Inspection	8/14/2008	4/6/2011
Pet Visit	9/25/2018	1/16/2019
Inspection	12/11/2017	5/21/2019
Ilicit Drug Possession	12/4/2013	7/21/2015
Pet Visit	10/13/2015	12/15/2017
Pet Visit	8/11/15	12/7/2017
Pet Visit	4/21/2014	2/21/2017
Pet Visit	4/25/2017	3/13/2018
Pet Visit	2/9/2016	2/22/2017

Pet Visit	8/10/2017	4/24/2018
Pet Visit	2/27/2018	8/2/2019
Inspection	7/14/2017	6/5/2018
Pet Visit	12/22/2017	9/6/2018
Pet Visit	4/29/2010	4/24/2012
Pet Visit	12/1/2014	10/21/2016
Out of State Discipline	7/7/2016	12/9/2016
Pet Visit	9/6/2016	7/14/2017
Out of State Discipline	5/31/2016	1/11/2017
Pet Visit	4/6/2016	11/2/2017
Out of State Discipline	7/12/2016	12/9/2016
Pet Visit	8/26/2016	8/1/2017
Out of State Discipline	12/9/2016	4/6/2017
Pet Visit	6/9/2017	3/7/2018
Pet Visit	2/2/2017	3/19/2019
Pet Visit	10/11/2018	4/16/2019
Out of State Discipline	6/19/2017	10/31/2017

Specifics

Lied about pre-surgical bloodwork being performed, but went ahead w surgery. Patient died "after" surgery. Did

Missed cranial mediastinal mass (case was clinical for 2 months) despite working for company advertising to be

Vet performed exploratory surgery for chronic vomiting and diarrhea, did not do bloodwork (or document PE find

Failed second premise inspection, inadequate record keeping for multiple patients, and only gave steroid injectio

Performed exploratory abdominal surgery without taking rads of suspected sewing needle foreign body. Wrote f

Failed to recommend hospitalization for kidney failure and pancreatitis; gave oral pred plus stronger steroids, an

Prescribed massive overdose of Bactrim, a dangerous drug. Failed to recognize, diagnose, and recommend foll

Extracted 8 teeth w/o owner consent. Failed to establish a VCPR. Altered, modified, and falsified medical record

Patient 1: Negligent for: failing to diagnose ear problem prior to treatment. performed unauthorized surgery. faile

Failed to make a written assessment of Pory's condition or tentative Dx. Failed to offer refferal or consultation to

Respondent submitted *altered* medical records to VMB that contained different information from complaintaint'

orthopedic Specialist Respondent only recommended "amputation" or "euthanasia" after apparent failed TPLO,

R failed to recognize and address feline renal dz from blood tests, and implications for tx of hyperthyroidism. Fai

There are so many facts, should I include them all? here are some examples: failed to interpret loss of detail and

Respondent failed to assess patient's renal health adequately before anesthetizing her. Respondent did not des

Blocked cat's emergency treatment was delayed 3 hours despite reassuring client that the obstruction would be

Did not administer post operative pain meds. Owner brought dog in for treatment, then left to "get money" and n

while respondent was not officially licensed to practice, the respondent performed veterinary medical duties inclu

Respondent did not have sufficient medical records. Dog came in for eye probelm but was too aggressive to exa

respondent did not renew license and eventually was inspected by the board. various violations.

dog brought in for euthanasia. when vet tried to sedate dog with intravenous injection, vet could not find vein. de

three consumer complaints first dog was attacked by Rottweiler and damaged assessment was insufficient, late

complaint was filed by a client with two cats. The first cat did not have an exam performed but x-rays were taken

dog fell out of pickup truck traveling 70 mph. respondent repaired fractured leg with IM pin and provided some in

Vet hired temporarily for SPCA neuter clinic. Used surgery room as breakroom, including putting shoes on surge

same as case 26

Failed to adequately treat sepsis initiated by foxtail foreign bodies and dog died at home, non-legible and missin

veterinarian spayed a service dog that was in heat. dog was left overnight without supervision and found dead in

inadequately shaved show dogs before surgeries and catheter placement. administered diluted vaccines agains

owner asked vet to declaw three of his cats. cats were not provided with pain control medications to last enough

dog brought in for vomitting and no bowel movements. GI foreign body diagnosed with rads. Exploratory laparot

Patient with suspected Immune Mediated Disease was not treated with immunosuppressive drug and patient die

#1: 3 puppies brought in for declaw removal. Paws on all 3 became swollen and infected. Client took to

Was Dealing drugs illegally. this is from petition to r

#1: consumer complaint initiated an investigation. dog presented for possible kennel cough and painful abdome

Vet developed parkinson's disease, did not write legible records, did not keep sufficient records. Allowed RVT to

#1: Ordered Venlafaxine for cat but took it herself. Told her manager she "couldn't function" without the med

Cat brought for teeth cleaning. Previous lab results from another vet showed renal insufficiency, but vet adminis

#1: Treated cat with hx of porto systemic shunt with vetalog injection. Client's regular vet wrote a witness sta

#1: Performed a lag screw fixation on the sacroiliac luxation and casting of the tibia on a patient hit by a

#1: Attempted to perform an OVH instead of a dental procedure to remove a canine tooth; Used a large dose

Case 1: Castrated a dog with an undescended left testicle and severed the urethra. Emergency Vet where do

#1 Spayed and declawed a cat who was only supposed to be declawed due to a record mix up; Owner discovere

Allowed unlicensed vet assistants to practice vet med (dental scaling, tooth extractions, and to induce anesthesi

DEA Inspection found violations: Did not report theft of controlled substances; Did not maintain her executed DE

#1: Performed home euthanasia for a dog without properly assessing it prior to the procedure. Dog suffered e

#1: Treated a 14 year-old cat with chronic renal insufficiency. Performed surgery to remove right lobe of thyr

#1: Dog rhinitis was not resolved at Respondent's clinic because potentially wrong diagnosis (bacterial in origi

Disciplined in FL in 2013 for failing to conduct a full exam of a dog before vaccination and allowing a vet tech to

#1: Administered too much Kenalog (0.25 cc) and DepoMedrol (90 mg) to a cat over about 2 months along wi

AZ placed license on probation for 2 years. Failed to provide professionally acceptable procedures by leaving a

#1 Puppy brought in for neuter, bloodwork showed healthy with anemia. Receptionist told owner puppy was

#1 2013: Failed to perform emergency surgery immediately, or place an E-collar on a dog who self-mutilated wh

15 year-old dog brought in w/ complaints of constipation, not urinating, and may have eaten some bedding. Res

AZ placed Respondent on 1 year probation in 2009 for failing to record a dog's heart and respiratory rates in the

Performed exploratory surgery even though contrast radiographs did not indicate need for surgery, left patient at

Claimed to have spayed patient but actually did not because Respondent claimed animal was already spayed. L

Board received two consumer complaints about a cat and a dog followed by one complaint about unsanitary cor

Arizona placed Respondent's license on probation for 1 year; Arizona Board found unprofessional conduct (faile

Intention_Status	Probation_Status	Probation_Duration_Yrs
Intentional	Yes	5
Unintentional	Yes	3
Intentional	Yes	3
Unintentional	Yes	3
Intentional	Yes	5
Intentional	Yes	5
Unintentional	Yes	3
Intentional	Yes	3
Intentional	Yes	5
Unintentional	Yes	3
Intentional	Yes	5
Unintentional	Yes	3
Unintentional	Yes	2
Intentional?	Yes	5
Intentional	Yes	5
Unintentional	Yes	3
Intentional	Yes	4
Intentional	Yes	3
Unintentional	Yes	4
Unintentional	Yes	3
Unintentional	Yes	3
Unintentional	Yes	4
Unintentional	Yes	5
Unintentional	Yes	3
Intentional	Yes	4
Intentional	Yes	3
Intentional	Yes	5
Unintentional	Yes	4
Intentional	Yes	5
Unintentional	Yes	3
Unintentional	Yes	3
Intentional	Yes	4
Intentional	No	
Intentional	Yes	4
Unintentional	No	
Intentional	No	
Intentional	Yes	5
Unintentional	Yes	4
Unintentional	Yes	5
Unintentional	Yes	5
Unintentional	Yes	4
Unintentional	Yes	4

Intentional	Yes	4
Unintentional	Yes	3
Intentional	No	
Intentional	No	
Intentional	Yes	3
Intentional	No	
Intentional	No	
Unintentional	No	
Unintentional	No	
Unintentional?	No	
Intentional	No	
Unintentional	Yes	4
N/A	No	
Unintentional?	No	
Intentional	No	
Intentional	No	
Unintentional?	No	

Individual_License_Suspended	Duration_License_Suspended_Days
No	
Yes	10
Yes	20
No	
Yes	30
Yes	45
No	
No	
Yes	60
No	
Yes	7
No	
No	
No	
No	
No	
Yes	3
No	
No	
No	
No	
Yes	15
Yes	60
No	
No	
No	
Yes	30
No	
Yes	30
No	
No	
Yes	15
No	
Yes	
No	
No	
Yes	45
Yes	
Yes	10
Yes	120
Yes	15
Yes	14

Yes	7
Yes	10
No	
No	
Yes	30
No	
No	
No	
No	
No	
No	
Yes	30
No	
No	
No	
No	
No	

License_Revoked_or_Surrendered	Revocation_Stayed	Restitution_Paid
Yes	Yes	No
Yes	Yes	Yes
Yes	Yes	No
Yes	Yes	No
Yes	Yes	No
Yes	Yes	Yes
Yes	Yes	No
Yes	Yes	No
Yes	Yes	No
Yes	Yes	No
Yes	Yes	No
Yes	Yes	Yes
Yes	Yes	No
Yes	Yes	No
Yes	Yes	No
Yes	Yes	No
Yes	Yes	No
Yes	Yes	No
Yes	Yes	Yes
Yes	Yes	No
Yes	Yes	No
Yes	Yes	No
Yes	Yes	No
Yes	Yes	Yes
Yes	Yes	No
Yes	Yes	No
Yes	Yes	No
Yes	Yes	Yes
Yes	Yes	No
Yes	Yes	No
Yes	Yes	Yes
Yes	Yes	No
Yes	No	No
Yes	Yes	No
Yes	No	No
Yes	No	No
Yes	Yes	No
Yes	Yes	No
Yes	Yes	Yes
Yes	Yes	No
Yes	Yes	Yes
Yes	Yes	Yes

Yes	Yes	No
Yes	Yes	No
Yes	No	No
Yes	No	No
Yes	Yes	No
Yes	No	No
Yes	No	No
Yes	No	No
Yes	No	No
Yes	No	No
Yes	No	No
Yes	Yes	Yes
Yes	No	No
Yes	No	No
Yes	No	No
Yes	No	No
Yes	No	No

Amt_Restitution	Fined_Status	Amt_Fined	Amt_Investigation_Cost_Recovery
	Yes	2000	15000
2800	Yes	1000	7533
	Yes	1000	9600
	Yes	5000	25778.48
	Yes	1500	28355.5
1500	Yes	2000	5100
	Yes	1000	7367.5
	No		14981
	Yes	2000	12952.5
	Yes	2000	6732.5
	Yes	2000	3953
	Yes	2500	5937.5
	Yes	3000	13275
	No		25000
	No		16735
	Yes	1000	5235
	No		9797.5
	No		9339.25
3400	Yes	2500	7447
	Yes	5000	2610
	No		18162.25
	Yes	2500	9243.62
	Yes	1500	7510
2500	No		5730
	No		5000
	Yes	1500	8538.42
	Yes	1500	14659.75
1000	Yes	2500	4951
	Yes	5000	40000
	Yes	1000	13020
3959.96	No		5077.5
	Yes	2000	19,643.03
	Yes	5000	51280
	Yes	3000	13476
	No		0
	No		64456.25
	Yes	2500	22000
	No		10780
1000	Yes	1000	5999.4
	Yes	5000	56206.87
2500	No		10210
2022.13	Yes	1500	6680

	Yes	2500	3,337.50
	Yes	5000	23183.75
	No		13197
	No		12002
	Yes	2000	13039.95
	No		9144
	No		3095
	No		11505
	No		887.5
	No		10162.5
	No		1315
1443.62	Yes	1500	7819
	No		1200
	No		8113.12
	No		41703.75
	No		38302.29
	No		935

CE_Status	Tot_Amt_Hrs_CE
Yes	80
Yes	48
Yes	60
Yes	24
Yes	240
Yes	200
Yes	60
Yes	36
Yes	100
Yes	48
Yes	100
Yes	60
Yes	24
Yes	80
No	
Yes	36
Yes	60
Yes	60
Yes	96
Yes	20
Yes	72
Yes	96
Yes	300
Yes	48
No	
Yes	60
Yes	125
Yes	48
Yes	40
Yes	12
Yes	12
Yes	80
No	
No	
No	
No	
Yes	120
Yes	80
Yes	100
Yes	90
Yes	120
Yes	80

Yes	128
Yes	45
No	
No	
Yes	60
No	
No	
No	
No	
No	
No	
Yes	96
No	
No	
No	
No	
Yes	6

CS_Status	Tot_Amt_Hrs_CS	ET_Status
No		Yes
Yes	16	No
No		Yes
No		No
Yes	40	Yes
Yes	16	Yes
Yes	20	No
No		Yes
Yes	20	Yes
No		No
Yes	20	Yes
No		Yes
No		Yes
No		Yes
No		No
No		No
Yes	20	Yes
Yes	120	Yes
No		Yes
No		No
Yes	10	Yes
No		No
No		No
No		No
No		Yes
No		Yes
No		No
No		Yes
Yes	400	Yes
No		No
Yes	20	Yes
No		Yes
No		No
No		No
No		No
No		No
Yes	40	Yes
No		No
No		Yes
No		No
No		Yes
Yes	20	No

No		Yes
Yes	240	Yes
No		No
No		No
No		No
No		No
No		No
No		No
No		No
No		No
No		No
No		No
No		No
No		No
No		No
No		No
No		No

Tot_Amt_Hrs_ET	Served_to_Order_day	Ser_Ord_yrs	Initial_to_Order_day
4	541	1.5	1872
	402	1.1	1474
6	176	0.5	1657
	63	0.2	1490
40	400	1.1	925
40	371	1.0	906
	638	1.7	1903
10	473	1.3	1638
15	548	1.5	1895
	384	1.1	1191
20	382	1.0	1089
24	369	1.0	1049
16	547	1.5	1514
20	295	0.8	2043
	453	1.2	1173
	439	1.2	1630
20	338	0.9	1167
54	294	0.8	2605
16	454	1.2	1045
	150	0.4	499
8	340	0.9	906
	559	1.5	1959
	951	2.6	3000
	175	0.5	724
8	422	1.2	1114
5	287	0.8	984
	733	2.0	1705
16	397	1.1	922
10	367	1.0	1036
	574	1.6	1435
4	216	0.6	793
45	462	1.3	1843
	703	1.9	2231
	965	2.6	2794
	113	0.3	1554
	526	1.4	1434
25	594	1.6	2041
	794	2.2	1965
40	849	2.3	2268
	1037	2.8	2756
24	322	0.9	2355
	379	1.0	1563

40	257	0.7	942
24	521	1.4	2223
	326	0.9	1280
	258	0.7	1840
	726	2.0	2211
	690	1.9	2271
	155	0.4	1158
	311	0.9	917
	225	0.6	684
	575	1.6	1182
	150	0.4	1144
	340	0.9	1248
	118	0.3	3615
	271	0.7	771
	775	2.1	1858
	187	0.5	1146
	134	0.4	608

In_Ord_yrs	Settlement
5.1	Revoked, Stayed; Probation 5 yrs; premise pe
4.0	Revoked, Stayed; Probation 3 yrs; obey laws;
4.5	Revoked, Stayed; Probation 3 yrs; Suspensio
4.1	Revoked, Stayed; Probation 3 yrs; obey laws;
2.5	Revoked, Stayed; Probation 5 yrs; Obey All la
2.5	Revoked, Stayed; Probation 5 yrs; Obey All la
5.2	Revoked, Stayed; Probation 3 yrs; obey all lav
4.5	revoked, stayed, probation 3 years; continuing
5.2	revoked, stayed, probation 5 years; obey all la
3.3	R, S, Probation 3 yrs; OAL; QRI; CwPS ($100
3.0	R, S, Probation 5 yrs: OAL; QRI; CwPS (100/r
2.9	R, S, Probation 3 yrs; OAL; QRI; CwPS ($100
4.1	R, S, Probation 2 yrs; OAL; QRI; CwPS ($100
5.6	R, S, Probation 5 yrs; OAL; QRI; CwPS ($100
3.2	R, S, Probation 5yrs. obey all laws. quarterly r
4.5	r, s, probation three years. obey all laws. quar
3.2	r, s, Probation 4 yrs. obey all laws. quarterly re
7.1	revoked stayed probation 3 years. obey all lav
2.9	revoked stayed probation 4 years. obey all lav
1.4	revoked stayed probation 3 years. obey all lav
2.5	revoked stayed probation 3 years. obey all lav
5.4	revoked stayed probation for years. obey all la
8.2	revoked stayed probation 5 years. obey all lav
2.0	revoked stayed probation 3 years. obey all lav
3.1	revoked stayed probation 4 years. obey all lav
2.7	Revoked; Stayed; 3 years probation. cost reco
4.7	revoked stayed probation 5 years. actual susp
2.5	revoked stayed probation 4 years. cost recove
2.8	revoked stayed 5 years probation. cost recove
3.9	revoked stayed 3 years probation. cost recove
2.2	revoked stayed 3 years probation. cost recove
5.0	In 2016: Stay lifted and revocation imposed fo
6.1	Revoked; Fine ($5,000); Costs of Investigatio
7.7	Revoked.
4.3	Revoked.
3.9	Vet license was revoked. Tech's license was r
5.6	Revoked; Stayed; Probation 5 years; Obey All
5.4	Revoked; Stayed; Probation 4 years; Obey All
6.2	Revoked; Stayed; Probation 5 years; Obey All
7.6	Probation 5 years; Suspension-Individual Lice
6.5	Revoked; Stayed; Probation 4 years; Obey All
4.3	**Case 1:** Revoked vet license and premises lic

2.6	Revoked vet license and premises permit; Sta
6.1	Revoked Vet License and Premises Registrat
3.5	Surrender vet license and premises permit; Lo
5.0	Surrender vet and premise licenses; Lose all r
6.1	Revoked; Stayed; Probation 3 years; Actual S
6.2	Surrender veterinary premise license and vet
3.2	Surrender license; Lose all rights and privilege
2.5	Surrender license; Lose all rights and privilege
1.9	Surrender license; Lose all rights and privilege
3.2	Surrender license; Lose all rights and privilege
3.1	Surrender license; Lose all rights and privilege
3.4	Revoked; Stayed; Probation 4 years; Obey All
9.9	Surrender license; Lose all rights and privilege
2.1	Surrender license; Lose all rights and privilege
5.1	Surrender license; Lose all rights and privilege
3.1	Surrender vet license and premises registratic
1.7	Surrender license; Lose all rights and privilege

Tot_Cost_Hrs_CE_ET_CS
84
64
66
24
320
256
80
46
135
48
140
84
40
100
0
36
100
234
112
20
90
96
300
48
8
65
125
64
450
12
36
125
0
0
0
0
185
80
140
90
144
100

168
309
0
0
60
0
0
0
0
0
0
96
0
0
0
0
6

Tot_cost_dollars_Resti_Recov_fine
17000
11333
10600
30778
29856
8600
8368
14981
14953
8733
5953
8438
16275
25000
16735
6235
9798
9339
13347
7610
18162
11744
9010
8230
5000
10038
16160
8451
45000
14020
9037
21643
56280
16476
0
64456
24500
10780
7999
61207
12710
10202

5838
28184
13197
12002
15040
9144
3095
11505
888
10163
1315
10763
1200
8113
41704
38302
935
