# Supplementary material for: Insights From Veterinary Disciplinary Actions in California 2017–2019
Source: Front Vet Sci. 2021 Dec 23;8:786265. doi: 10.3389/fvets.2021.786265 (PMC8734430; doi:10.3389/fvets.2021.786265)
Supplement: Supplementary Data 2 — Department of Consumer Affairs, Veterinary Medical Board, Consumer Complaint Form. [file Data_Sheet_2.PDF]

BUSINESS, CONSUMER SERVICES AND HOUSING AGENCY • GAVIN NEWSOM, GOVERNOR
DEPARTMENT OF CONSUMER AFFAIRS • VETERINARY MEDICAL BOARD
1747 North Market Blvd., Suite 230, Sacramento, CA 95834-2978
P (916) 515-5220 | Toll-Free (866) 229-0170 | www.vmb.ca.gov

# CONSUMER COMPLAINT FORM

## 1. COMPLAINT REGISTERED AGAINST - *please provide all information requested*

| | | | |
|---|---|---|---|
| NAME COMPLAINT AGAINST | | PHONE | |
| HOSPITAL/BUSINESS NAME | | | |
| HOSPITAL/BUSINESS ADDRESS | CITY | STATE | ZIP |

## 2. PERSON REGISTERING COMPLAINT - *please provide all information requested*

| | | |
|---|---|---|
| NAME OF PERSON REGISTERING COMPLAINT | WORK PHONE | |
| ADDRESS | HOME PHONE | |
| CITY | STATE | ZIP |
| HAVE YOU COMPLAINED TO ANOTHER ORGANIZATION OR AGENCY? (PLEASE SPECIFY) | | |

## 3. DETAILS OF COMPLAINT - *please provide all information requested*

| | | | | |
|---|---|---|---|---|
| PATIENT'S NAME | BREED | AGE | DATE OF VISIT | |
| REASON FOR VISIT | | | CURRENT PHYSICAL CONDITION | |
| CONSULTING VETERINARIANS (BEFORE/AFTER) | | | PHONE | |
| HOSPITAL NAME | | | | |
| HOSPITAL ADDRESS | | CITY | STATE | ZIP |

*Emphasis is placed on providing necessary factual information rather than conclusions. Supplemental information such as copies of any receipts, bills, letters or forms received from the veterinarian or veterinary facility is helpful. A Witness Statement Form is also very helpful in making a determination.*

## 4. ADDITONAL DETAILS OF COMPLAINT - *attach additional sheets if necessary*

______________________________

______________________________

______________________________

______________________________

## 5. SIGNATURE AND DATE

Notice: Except for the name of the veterinarian, all information is voluntary, but failure to provide the requested information may delay or prevent the investigation of your complaint. As much information as possible should be provided in connection with the complaint. The information on this form will be used in part to determine whether a violation of state law has occurred. If a violation is substantiated, the information may be transmitted to other government agencies, including the Attorney General's Office.

Signature ______________________________ Date ____________________

# WITNESS STATEMENT FORM

| NAME | WORK PHONE | |
|---|---|---|
| ADDRESS | HOME PHONE | |
| CITY | STATE | ZIP |
| DATE INCIDENT OCCURED | | |

*Note: If you have any questions or need assistance, please contact the enforcement assistant at (916) 515-5220*

**UNDER PENALTY OF PERJURY, I MAKE THE FOLLOWING STATEMENT:**

SIGNATURE DATE

*Attach additional sheets if necessary*

Rev. 2/2019
